# Consumers Emotional Responses to Functional and Hedonic Products: A Neuroscience Research

**DOI:** 10.3389/fpsyg.2020.559779

**Published:** 2020-10-06

**Authors:** Debora Bettiga, Anna M. Bianchi, Lucio Lamberti, Giuliano Noci

**Affiliations:** ^1^Department of Management, Economics and Industrial Engineering, Politecnico di Milano, Milan, Italy; ^2^Department of Electronics, Information and Bioengineering, Politecnico di Milano, Milan, Italy

**Keywords:** emotions, functional product, hedonic product, neuroscience, physiological signals

## Abstract

Over the years, researchers have enriched the postulation that hedonic products generate deeper emotional reactions and feelings in the consumer than functional products. However, recent research empirically proves that hedonic products are more affect-rich only for some consumer segments or for specific consumption contexts. We argue that such inconsistency may derive from the nature of the emotions assessed that is strictly dependent on their empirical measurement and not from the mere existence of emotions themselves. Self-reported methods of evaluating consumer experience, on which prior studies are grounded, only assess conscious emotions the consumer can recognize and report, but not unconscious feelings, happening without individual awareness. The present work takes this challenge by conducting a laboratory experiment in which subjects are exposed to both a utilitarian product and a hedonic product. Physiological measures have been adopted to investigate unconscious emotional responses and self-reported measures to assess conscious emotions toward the products. Specifically, physiological data regarding the subjects’ cardiac activity, respiratory activity, electrodermal activity, and cerebral activity have been collected and complemented with a survey. Results confirm that both functional and hedonic products generate emotional responses in consumers. Further, findings show that when a consumer is exposed to a functional product, the physiological emotional responses are disassociated from the self-reported ones. A diverse pattern is depicted for hedonic products. We suggest an alternative explanation for the apparent lack of affect-rich experiences elicited by functional products and the need to reconsider emotional responses for these products.

## Introduction

As a large body of research documents, consumers’ evaluations of new products are not purely utilitarian but dependent on the emotions and involvement elicited by the offer (Bagozzi et al., 1999; Kempf, 1999; Allen et al., 2005; Hassenzahl, 2018). Emotional responses constitute predictable and impactful drivers of decision making with regularities in the mechanisms through which they influence product evaluation (Lerner et al., 2015; Bettiga and Lamberti, 2017). Research on product adoption and consumption mainly argues that such emotional responses are processed differently by individuals according to the mainly hedonic or utilitarian/functional nature of the product they are evaluating (Hoch and Ha, 1986; Batra and Ahtola, 1991; Kempf and Smith, 1998; Ren and Nickerson, 2019; Yang et al., 2020). Hedonic products refer to objects consumed mostly for affective or sensory fulfillment aim, while utilitarian products are connected to more functional and practical benefits (Woods, 1960; Strahilevitz and Myers, 1998; Huber et al., 2018). Although this distinction is less than unequivocal (Holbrook and Hirschman, 1982), there appears to be a consensus that a main utilitarian product affects differently cognition and emotions than a hedonic product (Kempf, 1999). Hedonic products have been argued as being more affect-rich than those consumed for utilitarian purposes (Pham, 1998; Malhotra, 2005; Baghi and Antonetti, 2017). Research affirms that they generate greater arousal (Kempf, 1999), pleasure, and engagement (Kivetz and Simonson, 2002; Zheng and Kivetz, 2009; O’Brien and O’Brien, 2010) than utilitarian ones.

Given that, academicians and marketers have proposed different communication approaches for the two product typologies, assuming that emotional communication would be more effective for a hedonic offer (Johar and Sirgy, 1991; Rossiter et al., 1991; Batra and Stephens, 1994). However, recent research sheds doubts on the inherent difference in the emotions generated by hedonic versus utilitarian products, showing that hedonic offer generates greater emotions only for some customer segments (Drolet et al., 2007) or for specific interaction modes (Liao et al., 2016). Other studies found that emotional responses (e.g., Henning et al., 2012) and consumer responses (Vila-López and Küster-Boluda, 2018) do not differ for hedonic and functional product evaluation.

We propose that such discrepancy may derive from the nature of the emotions assessed, which is strictly dependent on their empirical measurement. Research typically measures emotions through self-reported techniques, such as surveys (e.g., Henning et al., 2012; Liao et al., 2016). Self-reported techniques, however, have shown significant limitations when it comes to assessing human reactions to stimuli and purchase patterns (Groeppel-Klein and Baun, 2001; Ait Hammou et al., 2013). Consumers, indeed, are typically unable to describe their emotional processes, given the subconscious mechanisms of which individuals are not aware of and thus cannot report (Berridge and Winkielman, 2003; Ivonin et al., 2013). These methods can only catch what consumers report, so the conscious emotions that the consumer can recognize and describe. However, self-reported methods cannot measure the unconscious feelings and emotions the individual experiences but is not able to account. For these reasons, several researchers highlight the need to measure physiological unconscious emotions that go beyond the subjective felt ones (Oatley, 1992; Bagozzi et al., 1999; Chamberlain and Broderick, 2007).

This research attempts to address such concern by assessing both the physiological (unconscious) and the self-reported (conscious) emotional reactions generated by hedonic and utilitarian products, given the importance of emotions in product adoption and consumption (Chaudhuri et al., 2010). We examine the influence of the product nature (functional and hedonic) on a consumer’s affective reactions of pleasure, arousal, and involvement through both physiological techniques (electroencephalography, heart rate, breath rate, and skin conductance) and self-reported instruments. With this study, we aim at providing theoretical and empirical evidence on the conscious and unconscious emotional responses generated by hedonic and utilitarian products. Further, we provide implications of the use of physiological techniques in the assessment of a consumer’s experience with new products.

## The Role of Emotions in Product Evaluation

### Pleasure, Arousal, and Involvement

Emotions may have two conceptualizations: discrete emotions, identified as individual and basic entities such as happiness, surprise, and sadness; or global feelings, identified in the two dimensions of arousal and pleasure. The validity of discrete emotions has been called into question by several researchers, as their identification was considered incoherent and trivial (Chamberlain and Broderick, 2007). In light of such criticisms, research has mainly focused on the global feelings of pleasure and arousal (Mehrabian and Russell, 1974), enabling a variety of measurement techniques with better results than discrete emotions assessment (Chamberlain and Broderick, 2007).

Pleasure (or valence) reflects happiness and satisfaction, while arousal conveys excitement, stimulation, and bodily activation. From a physiological viewpoint, arousal is a central component of behavior and a driver of decision-making processes (Groeppel-Klein, 2005). Arousal reflects an active body reaction; thus, it is closely related to attention to relevant outside stimuli and their processing (Groeppel-Klein, 2005) and has been acknowledged as a direct expression of involvement (Chaudhuri, 2002). Heightened arousal, indeed, has been found as a consequence of product involvement (Mitchell, 1980). The two emotional reactions, as suggested by literature (Chaudhuri, 2002; Groeppel-Klein, 2005), may be indeed strongly connected. Physiologically, involvement is identified as “the ability to focus on certain aspects of the environment while ignoring others” (Venkatraman et al., 2015, p. 438). It reflects the intrinsic interest and needs gratification that the consumer searches in the product (Zaichkowsky, 1985; Mittal and Lee, 1989; Batra and Ahtola, 1991). Arousal can have a positive or a negative valence: for instance, an individual showing high arousal can be either positively excited or highly irritated or upset. This view has been widely confirmed through empirical studies (Baker et al., 1992; Ward and Barnes, 2001) that showed the distinct nature of the arousal–relaxed and pleased–unpleased dichotomy and highlight the necessity to measure both dimensions to gather a complete understanding of consumer reactions.

### Measuring Emotions

The existence of an emotional state can be inferred by the means of physiological measures, self-report measures, or behaviors (Lang, 1968; Öhman, 1986). Marketing and consumer behavior research traditionally adopted self-reported measures to assess emotions, such as unipolar or bipolar scales on surveys (e.g., Mehrabian and Russell, 1974). A review of the main self-reported methods used in research is presented by Chamberlain and Broderick (2007). However, self-reporting may not reflect the real feelings that consumers experience (Ait Hammou et al., 2013) as individuals are typically unable to describe their emotional processes given the subconscious components that they cannot take into account (Kihlstrom, 1990, 1992; Kihlstrom et al., 2000; Berridge and Winkielman, 2003; Smith and Lane, 2016; for a review, see Robinson and Clore, 2002). Emotional responses, indeed, can be consciously experienced when they are generated by the identification of the eliciting cause. This happens through the recognition of the product that generates the emotions—e.g., a man pleased by a new pair of beautiful shoes (Kihlstrom, 1990). Or they can be unconscious when they are consciously experienced but without identification of the eliciting cause (misattribution) or generated but not consciously recognized (Kihlstrom et al., 2000). This is, for instance, the case of a consumer feeling anxious while using a new laptop but without knowing why. Despite the fact that prior research mostly failed to assess such unconscious emotions, it is widely acknowledged that most of the feelings that determine thought and behavior occur without awareness (Zaltman, 1997). While self-reported emotions are cognitive evaluations made *a posteriori*, physiological measures are not affected by the cognitive influences (Figner and Murphy, 2011). These measures can unveil the unconscious reactions of consumers to stimuli (Figner and Murphy, 2011) for which individuals are typically not aware of and hence not able to fully explain and report them (Fortunato et al., 2014). Physiological techniques have been confirmed successful in predicting consumer responses in a variety of contexts (Guixeres et al., 2017; Brás et al., 2018; Lin et al., 2018b; Sung et al., 2019).

## Emotions Toward Hedonic and Utilitarian Products

Research conventionally makes a distinction between hedonic and utilitarian products (Kempf, 1999; Kim and Morris, 2007; Lin et al., 2018a; Amatulli et al., 2020). Hedonic products are consumed mostly for affective or sensory fulfillment aim, while functional products for utilitarian goals (Woods, 1960; Strahilevitz and Myers, 1998; Kivetz and Simonson, 2002; de Witt Huberts et al., 2014; Lu et al., 2016). Hedonic goods are associated with fun, pleasure, and excitement (Khan et al., 2004). Typical examples of such products are perfumes, flowers, luxury watches, and sports cars. Utilitarian goods are primarily instrumental, and consumption is driven by functional aspects, such as for detergents, home security systems, or personal computers (Holbrook and Hirschman, 1982; Strahilevitz and Myers, 1998; Wertenbroch and Dhar, 2000).

Hedonic and utilitarian products have been associated with different consumer reactions and behaviors (Holbrook and Hirschman, 1982; Batra and Ahtola, 1991). Research has quite consistently argued that feelings are weighed more heavily under hedonic than utilitarian consumption goals (Pham, 1998; Malhotra, 2005). Products consumed for hedonic purposes have been acknowledged as more affect-rich and evoking feeling-based evaluations (Pham, 1998; Kempf, 1999; Malhotra, 2005). Research argued that hedonic product experiences lead to stronger emotional responses of arousal (Kempf, 1999; Fiore et al., 2005), pleasure, and engagement (Kivetz and Simonson, 2002; Zheng and Kivetz, 2009; O’Brien and O’Brien, 2010) than utilitarian ones. As a result, researchers and practitioners suggested different communication strategies according to the nature of the product marketed, assuming that affective communication evokes more positive consumer responses for a hedonic offer (Johar and Sirgy, 1991; Rossiter et al., 1991; Batra and Stephens, 1994). Emotional and value-related communication has been claimed as more relevant for such products (Johar and Sirgy, 1991; Rossiter et al., 1991), and ad liking seems connected to ad recall for hedonic but not for utilitarian objects (Youn et al., 2001). Overall, research moved toward the conclusion that the use of emotional appeal is desirable for hedonic products, while for utilitarian ones, it is not advised (Rossiter et al., 1991; Youn et al., 2001).

However, recent studies shed doubts on the inherent difference in the emotions generated by hedonic versus utilitarian products. Drolet et al. (2007) found that young adults have more positive attitudes toward and better recall of affective ads for hedonic products than utilitarian ones (for which rational ads work better), but the elderly have better recall and more positive attitude toward affective ads irrespective of the nature of the product advertised. Liao et al. (2016) proved that hedonic products, presented through an online interface, generate higher pleasure than utilitarian products but only in specific interaction conditions. They detected no differences, however, in the arousal dimension. Similarly, Sharma and Chan (2017) found mixed evidence on the moderating effect of product nature on counterfeit product purchase behaviors. Henning et al. (2012) and Bettiga and Lamberti (2018) established that emotions are relevant to both hedonic and functional product evaluations.

Such inconsistency in results may be generated by the empirical assessment of the emotions themselves, typically measured through self-reported techniques, by asking consumers to report the feelings they experienced. When interacting with hedonic products, indeed, consumers may devote higher attention to the emotional outcome of the consumption episode and emotions elicited by such interaction (Neelamegham and Jain, 1999). For certain products, such as movies, the emotional outcome may itself be the objective (Neelamegham and Jain, 1999). As emotions are perceived to be more important for hedonic consumption motives, consumers may pay more attention to their emotional reactions for hedonic products (Pham, 1998). Even when emotional responses are elicited similarly in both hedonic and utilitarian consumption, consumers are more likely to infer that their emotional responses have been elicited by the product itself (and not by other contextual elements) only for hedonic consumption (Henning et al., 2012). Thus, it is plausible that unconscious emotions are translated in consciously experienced emotions for hedonic products only, where there is an identification of the eliciting cause. Hence, we argue that the attribution of emotions mainly to hedonic products in mainstream research (e.g., Kempf, 1999) may be due to the conscious recognition and reporting of such emotions by consumers and not by the magnitude of real emotions experienced. For utilitarian products, conversely, consumers may not translate unconscious emotions in conscious, thus reportable, feelings, regardless of the real emotions experienced.

Grounding on this discussion, we expect that both functional and hedonic products generate emotions. However, such emotions may not be consciously recognized in utilitarian consumption scenarios, thus generating a misalignment between unconscious emotions and conscious emotions. Conversely, we expect that the unconscious emotions elicited by a hedonic product may be recognized at the conscious level. In other words, we posit that consumers do experience and are able to report emotions for hedonic products. Hence, we expect an alignment between physiological unconscious emotions and self-reported, thus conscious, ones. On the contrary, for functional products, consumers do experience emotions but are not able to report them; hence, we expect a misalignment between physiological and self-reported emotions. More formally, we propose:

**H1:** *There are no significant differences between the emotional reactions of (H1a) arousal, (H1b) pleasure, and (H1c) involvement generated by hedonic and functional products.*

**H2:** *For functional products, conscious and unconscious emotions of (H2a) arousal, (H2b) pleasure, and (H2c) involvement are misaligned.*

**H3:** *For hedonic products, conscious and unconscious emotions of (H3a) arousal, (H3b) pleasure, and (H3c) involvement are aligned.*

## Materials and Methods

### Laboratory Experiment

A laboratory experiment was conducted inside a university bioengineering laboratory to evaluate a consumer’s responses toward a functional product and a hedonic product. The experimental base was composed of 21 subjects (14 males, 7 females) aged between 22 and 25 years old. The narrow age range assures the full comparability of physiological data collected, as they may vary with age (e.g., Hayano et al., 1990). The sample size is in line with prior experiments adopting biometric measures (Vecchiato et al., 2012). Demographic statistics are reported in Table 1. We used a body scale as a functional product and an MP3 player as a hedonic product, according to prior research (Bettiga et al., 2017a), showing a significant difference in the perceived nature of the two products. The brands chosen were unfamiliar in the market where the experiment was conducted to avoid extra-experimental sources of variance caused by brand-related attitude. Half of the subjects were exposed firstly to the functional product and then to the hedonic product. The other half of the subjects were exposed firstly to the hedonic product and then to the functional product. Randomization was necessary to avoid possible confounding effects.

**TABLE 1:** Demographic statistics.

**Sex**	Male 66%	Female 33,3%	
Age	Min 22	Average 23.6	Max 25
Study title	Bachelor’s degree 85.7%	Master’s degree 14.3%	
Employment	Students 95%	Nonstudents 5%	
Nationality	Italian		

*N = 21.*

All volunteers were welcomed and briefly explained what the experimental protocol would have consisted of and were told they could withdraw from the experiment at any time. Subjects were instructed that the study aimed to assess their evaluation of commercial products and that, after the product examination, they will be asked to complete a questionnaire to record their evaluation. This procedure, according to prior research (Kempf and Smith, 1998), serves to prime the respondents to engage in product evaluation. During the study, subjects were comfortably seated in front of a PC monitor used for stimuli delivery. During the whole experiment, we collected the subjects’ cardiac activity (electrocardiogram, ECG), respiratory activity, electrodermal activity (EDA), and brain activity (electroencephalographic signals, EEG) to detect their unconscious emotions of arousal, pleasure, and involvement. Further, we assess, through a questionnaire, self-reported measures of arousal, pleasure, and involvement. The use of both physiological and self-reported methods allows testing the existence and the alignment/misalignment of conscious and unconscious emotions. To assure the absence of any kind of social influence or disturbance, the experiment was performed on one subject at a time. The study was organized into four consecutive phases:

I. A 2-min-long phase of mathematical calculations aimed at increasing the participants’ level of stress.
II. A 3-min-long phase of rest, in which the volunteers were asked to stare at a picture and to relax. This procedure was necessary to assess a baseline for each respondent, a condition against which physiological changes during the experiment can be compared.
III. The browsing of an *ad hoc* webpage displaying information and pictures about a commercial product (a functional product and a hedonic product). This phase could last at most 5 min.
IV. The filling of a questionnaire to collect self-reported measures about the product experience, plus individuals’ demographic information.

The first three phases of the experimental protocol were implemented using the Matlab software (Matlab version R2014a, The MathWorks, Inc.). During phase III, all participants could freely browse each webpage for the time they needed within the 5-min-long duration of this phase. The 5-min time limit serves to minimize underexposure or overexposure to one type of experience and yet provide enough duration not to affect the inherent advantages associated with the virtual experiences (Daugherty et al., 2008). The provision of *ad hoc* pages assures that (i) respondents do not browse other webpages or get distracted by web banners and pop-up as it may happen while browsing real webpages and (ii) we could design identical webpages for the two products under test to avoid differences in the virtual experience. Product webpages had the same layout, colors, and interaction possibilities. Each webpage had four links: home page, image, information, and supplementary information. Each subject could visit each link all the time he or she wanted.

### Measurements

#### Confounding and Manipulation Variables

We measured two confounding variables: product perceived diagnosticity and product perceived nature. Product perceived diagnosticity represents the consumer’s perception of the ability of a trial to help him or her understand the product. Diagnosticity should be perceived equal for the two products (i.e., both virtual interactions offer representative, credible evidence of the product and its attributes). This check guarantees that both webpages offer enough informative experience, as diagnosticy is able to affect product experience processing (Hoch and Ha, 1986; Kempf and Smith, 1998). Product diagnosticity was assessed via a single-item scale by asking, “Overall, how helpful would you rate the website navigation you just had in judging the quality and performance of the product?” Responses were assessed on a 1–7 scale with the endpoints labeled “not helpful at all” and “extremely helpful” (Kempf, 1999).

Secondly, to confirm that our manipulation of product nature was successful, we asked participants to rate each product on a 7-point scale according to perceived functional versus hedonic characteristics. This approach is similar to the one adopted in prior studies (Kempf, 1999). Specifically, we asked, “Would you characterize the [product] as primarily a functional product or an entertainment/enjoyable product?” with a 7-point scale, with 1 being “primarily for functional use” and 7 being “primarily for entertainment use” (Kempf, 1999).

#### Physiological Measures

Physiological signals were collected during the whole experiment. Specifically, we collected data regarding the subjects’ cardiac activity (electrocardiogram, ECG), respiratory activity, and EDA using a unique device (ProComp Infiniti encoder, Thought Technology Ltd., Quebec, Canada) to assess the subject’s level of arousal. The ECG signal was acquired using three disposable electrodes placed on the volunteer’s chest (the negative and the ground electrodes were placed on the right and left shoulder, respectively, while the positive electrode was placed above the right iliac spine). The respiratory activity was measured using a sensorized belt to be fastened around the participant’s chest. The EDA signal was acquired using two electrodes sewn inside Velcro straps to be fastened around the second and third fingers of the participant’s nondominant hand. The EDA signal is a measure of the skin’s ability to conduct electricity and represents changes in the sympathetic nervous system. A broad consensus exists among researchers that have recognized changes in heart rate, breath rate, and EDA as a reflection of changes in the level of activation generated during an emotional episode (Dawson et al., 2007; Sequeira et al., 2009; Dawson, 2011; Boucsein, 2012).

Furthermore, we collected data regarding the subjects’ brain activity (electroencephalographic signals, EEG) using the SD LTM EXPRESS headbox (Micromed S.p.A, Mogliano Veneto, Italy) and a 61-channel head cap. Specifically, the cap was placed above the subject’s head, and a conductive gel was used to acquire the brain signals from 28 channels (Fp1, FPz, FP2, F7, F3, Fz, F4, F8, T3, C3, Cz, C4, T4, T5, P3, Pz, P4, T6, O1, O2, AF7, AF3, AF4, AF8, F5, F1, F2, F6). We selected these specific channels as they enable the measurement of quantitative indexes of attention and pleasantness (Vecchiato et al., 2010, 2011, 2012).

#### Physiological Data Elaboration

All physiological data were elaborated using appropriate methods of signal processing following the relevant literature. As physiological measures for three subjects were not properly recorded, we did not consider these subjects in further elaboration, proceeding with analysis on a sample of 18 (11 males, 7 females) subjects. The elaborated data were then used to compute quantitative indexes to be correlated with the results of the questionnaire. Specifically, the heart rate variability (HRV) was obtained from the ECG signal as the time series of the heartbeat time intervals (Pan and Tompkins, 1985). The combined effect of cardiac and respiratory activity was taken into account using a bivariate time-variant autoregressive model (Barbieri et al., 1997, 2002; Mainardi et al., 1997), from which a quantitative feature (PSDc/r) describing the amount of HRV signal driven by respiration was computed (Bianchi et al., 1990). HRV and PSDc/r were obtained on a beat-to-beat base; the values were normalized by subtracting their average value during the baseline and by dividing by their standard deviation during the baseline. For this purpose, we used the last minute of the 3-min-long baseline phase. After that, the normalized HRV and PSDc/r beat-to-beat values were averaged across phase III to obtain one single value for each index. HRV_*III*_ and PSDc/r_*III*_ are the obtained quantities analyzed in this study. Both the ECG and the respiration signals were processed using custom algorithms developed in Matlab.

The EDA signal was processed using the deconvolution method through Ledalab V3.4.9^1^, a Matlab-based software package that performs event-related analysis relative to events/marker and returns various parameters of the EDA phasic—fast component, indicating the emotion induced by the stimulus—and tonic—slow component, indicating the baseline state activity (Benedek and Kaernbach, 2010a,b). Three quantitative indices were computed from the EDA signal: the average tonic activity (EDA_T) during phase III, the integrated skin conductance response (ISCR) as the time integral of the phasic activity during phase III, and the maximum value of phasic activity (PhasicMax) during phase III. The EDA_T index was normalized by subtracting its average value during the last minute of the 3-min-long baseline phase. This normalization was not necessary for the ISCR and the PhasicMax indexes, as these values are not affected by the subject’s baseline.

The EEG signal was processed as explained in Vecchiato et al. (2012) to compute the attention (AI) and the pleasantness (PI) indices. Both indices were obtained from the brain signals measured above the frontal and prefrontal cortices (i.e., electrodes Fpz, AF3, F3, AF4, F4, Fz for AI, electrodes AF3, AF4, F3, F4 for PI), as the activity of neurons belonging to these areas has been correlated with attention (Klimesch, 1999; Aftanas and Golocheikine, 2001) and pleasure (Davidson, 2004; Vecchiato et al., 2011). As done in Vecchiato et al. (2012), the AI index has been reversed to have the activity of desynchronization pointing up. Therefore, an increase in the subject’s attention is marked by an increase in the AI index. As concerns the PI index, the pleasure toward the product is marked by positive values. AI and PI values were normalized by subtracting their average value during the baseline and by dividing by their standard deviation during the baseline. For this purpose, we used the last minute of the 3-min-long baseline phase. After that, the normalized AI and PI values were averaged across phase III to obtain one single value for each index.

#### Self-Reported Measures

Self-reported emotional responses of arousal, pleasure, and situational involvement were collected through the mean of a questionnaire using validated scales after browsing each website. Table 2 reports the expected correspondence between physiological and self-reported measures of emotions. According to prior research (Havlena and Holbrook, 1986; Kempf, 1999), we used the scale developed by Mehrabian and Russell (1974) to measure the arousal–quietness dichotomy. The arousal scale items used, listed in random order, were “excited–calm,” “stimulated–relaxed,” “aroused–unaroused,” “sluggish–frenzied,” “dull–jittery,” and “sleepy–wide awake.” Also, pleasure was measured with the Mehrabian and Russell (1974) scale. The specific questions for pleasure were six semantic differential items, randomly presented: “happy–unhappy,” “pleased–annoyed,” “satisfied–unsatisfied, “melancholic-contented,” “despairing–hopeful,” and “bored–relaxed.” Both scale items were introduced with this instruction (Bradley and Lang, 1994): “Each line on the page contains an adjective pair which you will use to rate your feelings about the product. Some of the pairs may seem unusual, but you will probably feel more one way about one side than another. So, for each pair, place a checkmark close to the adjective which you believe describes your reaction to the picture better. The more appropriate the adjective seems, the closer you should put your checkmark to it.” We measured situational involvement, a measure of the involvement and attention devoted to the product stimuli, through three 7-item Likert scales, randomly listed, asking: “I was absorbed intensely in examining the product presentation,” “I concentrated fully on viewing the product presentation,” and “My attention was focused on examining the product” (Webster and Ho, 1997).

**TABLE 2:** Expected correspondence between physiological and self-reported measures of emotions.

**Emotional response**	**Physiological instrument**	**Physiological measures (unconscious)**	**Self- reported instrument**	**Self-reported measures (conscious)**
Arousal	Electrodermal activity (EDA)	EDA_T ISCR PhasicMax	Survey	Arousal scale (Mehrabian and Russell, 1974)
	Respiratory activity and cardiac activity (ECG)	HRV_*III*_ PSDc/r_*III*_		
Attention/involvement	Electroencephalographic signals (EEG)	Attention Index (AI)	Survey	Situational involvement scale (SI) (Webster and Ho, 1997)
**Pleasure**	Electroencephalographic signals (EEG)	Pleasure Index (PI)	Survey	Pleasure scale (Mehrabian and Russell, 1974)

## Results

### Self-Reported Data Elaboration

We performed a reliability analysis for self-reported constructs by assessing Cronbach’s alpha (Table 3). Results showed that our constructs are all reliable, with the pleasure construct for the functional product slightly under the commonly suggested threshold of 0.7 (Hair et al., 2012). We verified the absence of common method bias, which may be an issue when self-reported questionnaires are used to collect answers from the same participant at the same time. It represents the variance that may be attributed to the measurement method rather than the constructs that the measures represent. We employed Harman’s single factor test (Podsakoff et al., 2003), which assesses the presence of common method bias by indicating whether a single latent factor offers an acceptable alternative explanation of the analysis. Results show that the single factor was explaining less than 50% of the variance; thus, we concluded that common method bias does not represent a significant threat to the study.

**TABLE 3:** Reliability analysis for self-reported constructs.

	**Functional product**		**Hedonic product**	
**Construct**	No. of items	Cronbach’s alpha	No. of items	Cronbach’s alpha
Arousal	6	0.847	6	0.714
Situational involvement	3	0.743	3	0.886
Pleasure	6	0.612	6	0.742

**N* = 18.*

### Manipulation and Confound Variables Check

We checked if our manipulations were successful by asking subjects to report the perceived product nature. As expected, subjects perceived the body scale as a functional product with a mean of 1.44 and the MP3 player as a hedonic product with a mean of 5.17. These means were significantly different (*t* = −8.63, *p* < 0.001), confirming the goodness of the manipulation. Regarding product perceived diagnosticity, we confirmed that no significant differences exist between conditions with a mean of 5.17 for the functional product and a mean of 4.67 for the hedonic product (*t* = 1.18, *p* = 0.244).

### Mean Comparison Between Hedonic and Functional Products

**FIGURE 1:**
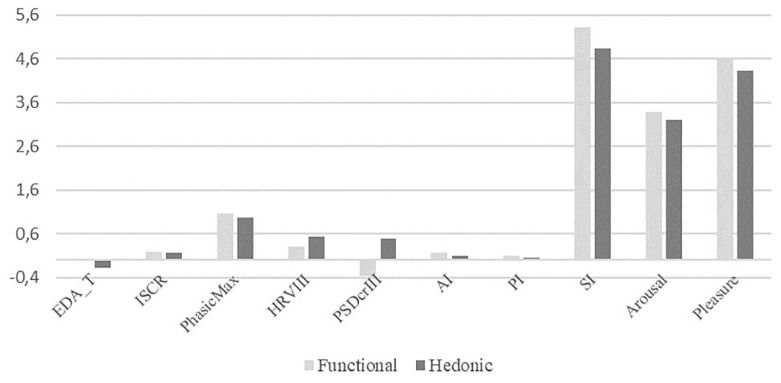
Mean comparison between hedonic and functional products.

We performed a *t*-test to check if the emotions generated by functional products differ significantly with the emotions generated by hedonic products. The results indicate that there is no statistically significant difference between the mean of both physiological and self-reported emotions of arousal, pleasure, and involvement for functional and hedonic products. The mean of each variable is reported in Figure 1; more detailed results are reported in Table 4. Thus, both products generate emotions of arousal, pleasure, and involvement in consumers at the physiological as well as self-reported level.

**TABLE 4:** Mean and standard deviation of physiological and self-reported measures.

***Product***		***Physiological measures***							***Self-reported measures***		
		EDA_T	ISCR	PhasicMax	HRV_III_	PSDcr_III_	AI	PI	SI	Arousal	Pleasure
*Functional*	Mean	0.01	0.19	1.07	0.31	–0.35	0.16	0.09	5.31	3.39	4.59
	SD	0.66	0.34	1.25	0.68	1.90	0.27	0.25	0.88	1.00	0.49
*Hedonic*	Mean	–0.18	0.16	0.98	0.53	0.49	0.09	0.06	4.83	3.21	4.33
	SD	0.37	0.23	1.06	0.76	2.33	0.14	0.15	1.32	0.86	0.60

*N = 18.*

### Correlation Analysis Results

A correlation analysis was performed between the indexes obtained from the physiological data and the declared ones. As the indexes obtained from the physiological data did not follow a normal distribution, the correlation analysis was performed using a two-tailed Spearman nonparametric statistical test with a significance level set equal to 0.05. Results are reported in Tables 5, 6 for the functional and the hedonic product, respectively.

**TABLE 5:** Correlation analysis for the functional product.

	**Emotional response**	**Sign of the value**		**EDA_T**	**ISCR**	**PhasicMax**	**HRV_III_**	**PSDc/r_III_**	**AI**	**PI**	**Arousal**	**SI**	**Pleasure**
Physiological measures	Arousal	Positive	EDA_T	1.00	0.61*	0.58*	0.32	0.14	0.38	0.11	–0.03	–0.01	–0.13
		Positive	ISCR	–	1.00	0.94*	0.00	0.35	0.72*	0.01	−0.48*	–0.29	0.01
		Positive	PhasicMax	–	–	1.00	0.17	0.31	0.79*	0.08	–0.39	–0.24	0.04
		Positive	HRV_III_	–	–	–	1.00	0.20	–0.03	–0.25	0.39	0.26	0.06
		Negative	PSDc/r_III_	–	–	–		1.00	0.11	−0.56*	–0.07	0.02	0.06
	Attention	Positive	AI	–	–	–	–	–	1.00	–0.03	–0.15	–0.24	0.31
	Pleasure	Positive	PI	–	–	–	–	–	–	1.00	–0.27	–0.49	0.12
Self-reported measures	Arousal	Positive	Arousal	–	–	–	–	–	–	–	1.00	0.50*	0.10
	Situational Involvement	Positive	SI	–	–	–	–	–	–	–	–	1.00	–0.04
	Pleasure	Positive	Pleasure	–	–	–	–	–	–	–	–	–	1.00

*Statistically significant correlations (*p*-value <0.05) are marked with an asterisk.*

**TABLE 6:** Correlation analysis for the hedonic product. (0.05) are marked with an asterisk.

	**Emotional response**	**Sign of the value**		**EDA_T**	**ISCR**	**PhasicMax**	**HRV_III_**	**PSDc/r_III_**	**AI**	**PI**	**Arousal**	**SI**	**Pleasure**
Physiological measures			EDA_T	1.00	0.25	0.35	–0.04	–0.05	–0.10	–0.23	0.15	0.42	0.06
	Arousal	Positive	ISCR	–	1.00	0.92*	0.38	–0.12	0.67*	–0.04	0.14	0.00	0.45
		Positive	PhasicMax	–	–	1.00	0.31	–0.18	0.66*	–0.15	0.11	0.04	0.45
		Positive	HRV_III_	–	–	–	1.00	−0.72*	0.46	–0.10	0.36	0.42	0.27
		Positive	PSDc/rV_III_	–	–	–		1.00	–0.27	–0.07	−0.48*	−0.54*	–0.41
	Attention	Negative	AI	–	–	–	–	–	1.00	0.17	–0.07	–0.21	0.36
	Pleasure	Positive	PI	–	–	–	–	–	–	1.00	–0.02	–0.11	0.25
	Arousal	Positive	Arousal	–	–	–	–	–	–	–	1.00	0.55*	0.15
	Situational Involvement	Positive	Pleasure	–	–	–	–	–	–	–	–	1.00	0.37
	Pleasure	Positive	SI	–	–	–	–	–	–	–		–	0.37

*Statistically significant correlations (*p*-value < 0.05) are marked with an asterisk.*

#### Correlation Analysis Between Physiological Data

For both products, the ISCR and the maximum value of phasic activity (PhasicMax) of the EDA signal show a statistically significant positive correlation, as they are both quantitative descriptors of the EDA phasic component, indicating unconscious arousal. Furthermore, both parameters show a significant positive correlation with the attention index (AI) obtained from the EEG signal. This result could suggest that the subject’s attention, as quantified using information from the brain signals, increases with increasing physiological arousal, as quantified using information from the EDA signal.

#### Correlation Analysis Between Self-Reported and Physiological Data

The most significant results in the correlation analysis between self-reported and physiological data have been found for arousal and involvement. Specifically, for the hedonic product, the quantitative parameters obtained from the cardiac and respiratory signals show statistically significant correlations with the subjective responses. The combined effect of cardiac and respiratory activity (PSDc/r) negatively correlates with the self-reported situational involvement (SI) and with the self-reported arousal. The negative sign of the correlation is that high values of the PSDc/r identify a relaxing condition, while low values identify a stressful condition. Thus, the more engaging experience relates to high reported situational involvement and arousal, showing alignment between physiological and self-reported arousal for the hedonic product. On the contrary, for the functional product, a statistically significant correlation has been detected between physiological arousal (measured through ISCR) and self-reported arousal. As both parameters measure the engagement of the individual, the negative sign of the correlation indicates that the self-reported arousal is misaligned with the physiological arousal for the functional product.

## Discussion

Our results suggest that the ability to generate emotions and feelings in the consumer is not the distinguishing mark between the two product typologies. The discriminant seems, however, to lie in the connotation associated *a priori* to the offer. In our study, consumers, indeed, declared that the two products have either a functional nature or a hedonic nature. We can infer that such awareness induces to report arousal and engagement only for the products perceived to be hedonic. This is in line with recent studies, affirming that when interacting with hedonic products, consumers may devote greater attention to the emotions elicited by such interaction (Pham, 1998; Neelamegham and Jain, 1999) and are more likely to infer that their emotional responses have been provoked by the product itself (Henning et al., 2012). Hence, the same distinction between hedonic and functional products may not lie in their inherent nature but in the rationalization of their consumption. A product may be classified as functional when individuals recognize its utilitarian value but not the emotional one. Similarly, a product may be perceived as hedonic because the individual recognizes its entertainment and emotional value in the absence of perceived utilitarian value. Consumers may justify their consumption of hedonic products by embedding them with emotional values and of functional products by recognizing in them prominent utilitarian values. Such findings would confirm what has been proposed by Addis and Holbrook (2001) regarding hedonic objects: “subjectivity might be more than just a filter, but an actualizing creative force that molds the object (via a perception of it) so as to shape the resulting consumption experience (including variable emotional reactions) in ways that defy rational analysis”(Addis and Holbrook, 2001, p. 60). If the subjectivity of the consumer evaluation is the distinguishing mark, the same ambiguity in the classification of some products as either functional or hedonic would be solved. Indeed, such discriminant does not lie in the product itself but in its interpretation by the individual. For instance, coffee may be perceived as functional if the individual consumes it because of stimulation. But it can be perceived as hedonic if the consumption is driven by the sensory enjoyment of coffee aroma. In the first case, post-consumption, the consumer will be more prone to recognize the energizing boost of the coffee, while in the second case its sensory attributes.

## Conclusion

This research aims at providing theoretical and empirical evidence on the conscious and unconscious emotional responses generated by hedonic and utilitarian products. We examine the influence of the product nature (functional and hedonic) on a consumer’s affective reactions of pleasure, arousal, and involvement through both physiological techniques (electroencephalography, heart rate, breath rate, and skin conductance) and self-reported instruments.

Findings show that functional and hedonic products both generate emotional responses in consumers, confirming H1a, H1b, and H1c. Neither the self-reported measures of arousal, pleasure, and involvement nor the physiological ones were showing any difference between the two product typologies, contrary to what has been argued by mainstream research (e.g., Pham, 1998; Malhotra, 2005; O’Brien and O’Brien, 2010) but in line with recent studies (Vila-López and Küster-Boluda, 2018). Further, findings of this study show that when a consumer is exposed to a functional product, the physiological emotional responses are disassociated from the self-reported ones, indicating that unconscious emotions generated by functional products may not be consciously recognized. Findings hold for arousal, pleasure, and involvement, confirming H2a, H2b, and H2c. Specifically, for arousal, we found that EDA_T and Phasic Max measures were not significantly correlated with self-reported arousal, while ISCR was negatively correlated. Similarly, physiological pleasure (PI) and physiological attention (AI) were not correlated with the respective self-reported measure of pleasure and situational involvement.

Conversely, for the hedonic product, results show a significant correlation between self-reported and physiological arousal, suggesting that unconscious arousal has been recognized at the conscious level by individuals, according to our H3a. In particular, our findings show that self-reported arousal significantly correlates with physiological arousal measured through the combined effect of cardiac and respiratory activity (PSDc/r). However, we found no correlation between self-reported and physiological pleasure and involvement, thus not confirming H3b and H3c. Such dissimilarity in findings may explain the recent controversial results of some works (e.g., Liao et al., 2016) that depict how hedonic offer may generate different outputs in terms of pleasure and arousal. Hedonic products, indeed, may generate higher pleasure than functional products only in some interaction modes (Liao et al., 2016), underlying the need of further research on this emotional reaction (Alba and Williams, 2013).

It should be noticed that, as suggested by literature (Chaudhuri, 2002; Groeppel-Klein, 2005), arousal and involvement show to be strongly connected, being self-reported measures of arousal and situational involvement positively correlated for both the hedonic and the functional products. The same holds for physiological measures, where the subject’s attention (AI), as quantified using information from the brain signals, significantly increases with increased activation of the sympathetic nervous system, as quantified using information from the EDA signal (ISCR and PhasicMax). Interestingly, for the hedonic consumption scenario, such connection is evident in the correlation between self-reported and physiological measures of arousal. Here physiological arousal measured through PSDc/r correlated with both self-reported arousal and self-reported involvement, providing further confirmation to H3a. Thus, our findings provide support to the assumption that, even when arousal is elicited similarly in both hedonic and utilitarian consumption, individuals are more likely to consciously recognize their emotional responses for hedonic consumption only.

## Research and Managerial Implications

The study contributes to research in three main directions. First of all, it warrants new findings to research on functional and hedonic consumption by depicting the different emotional reactions that consumers show while interacting with such product typologies. Despite numerous writers suggesting that hedonic, but not functional, products provide emotional experiences to individuals, we determine that functional products elicit emotional feelings in the consumer in the same extent as hedonic products.

Secondly, results show that functional products elicit unconscious emotions in consumers that, however, are not consciously recognized. Conversely, unconscious arousal and involvement generated through hedonic consumption are consciously recognized by consumers and thus can be reported. This finding may explain the lack of emotional reactions toward functional products found in prior research. The self-reported methods used in all prior studies (e.g., Kempf, 1999) may have been inadequate to detect the emotional reactions toward functional products. As we show in this work, even if consumers feel aroused and engaged, they can report it only for the hedonic product and not for functional ones. Thus, we propose an alternative explanation to the apparent lack of affect-rich experiences elicited by functional products that research has neglected. Results stress the need to reconsider emotional reactions for utilitarian products as well. From a managerial perspective, this provides new weight to emotional communication strategies, as marketers may evaluate the opportunity to convey emotional and visceral messages to promote utilitarian products above hedonic ones. This is especially relevant in light of the aforementioned role of subjective evaluations of the product nature, more than objective classification, for which marketing communication plays a major role.

This leads to the third contribution of this work, which is methodological. The study, indeed, shows that traditional instruments, such as surveys, and physiological analyses provide complementary information about the feelings and emotions generated by products. The former detect the conscious emotions for which the consumer is aware and thus can report. The latter provide information about the unconscious emotional reactions that research acknowledges as powerful drivers of decision-making (e.g., Kihlstrom et al., 2000; Ivonin et al., 2013). Thus, this research provides an initial step toward using physiological responses to deeply evaluate a consumer’s experience with new products. In line with such results, we suggest that marketers and product managers should adopt physiological methods in combination with self-reported ones to properly assess the experience evoked by their products both earlier along the new product development process and at the end of the process to develop marketing communication accordingly.

## Limitations and Future Research

The findings of this work are expected to be particularly robust due to the deployment of an experimental study in a laboratory setting using two different assessment methods: self-reported measures and physiological ones. Moreover, several physiological tools (electroencephalography, electrocardiogram, respiratory activity, and EDA) have been used to assess the emotional reactions of consumers. However, the choice of the laboratory experiment as the empirical setting, despite the fact that it provides higher internal validity being not affected by external influences, is lower in external validity. The artificiality of the setting, indeed, may have produced unnatural behaviors or reactions in consumers that do not reflect real-life behaviors. Thus, it limits generalizability to real environments in which consumers interact with products.

Additionally, future research is needed to replicate and extend our findings. In this work, we tested two electronic devices that, despite having been validated as representative of the hedonic and functional typologies (Bettiga et al., 2017a), pertain to a specific product category. Thus, we suggest replicating our study on different product categories to understand if differences in emotional reactions may occur. It would be particularly interesting to deploy such a study on product categories that are balanced in terms of functional and hedonic features to explore the role of consumer subjective evaluation in their classification and their subsequent emotional responses toward consumption. Similarly, the empirical test has been conducted on consumers of a restricted age range. Even if a reduced age range is necessary for physiological experiments, to assure the comparability of data collected, a replication of such empirical study on other consumer segments may provide additional information on the consumer’s emotional patterns.

Finally, we measured emotions through physiological measures and self-reported scales, revealing that both measures are necessary and showing that conscious and unconscious emotions are, in some instances, not aligned, in others positively correlated. However, it would be interesting if future studies could investigate the extent of such relationships and additional factors that may affect them.

## Data Availability Statement

The raw data supporting the conclusions of this article will be made available by the authors, without undue reservation.

## Ethics Statement

Ethical review and approval was not required for the study on human participants in accordance with the local legislation and institutional requirements. There are several reasons why an explicit approval by an Ethics Committee was not necessary: the participants are adults, the research does not involve vulnerable subjects, the participation in the study is voluntary, it is a minimal risk research, all data collected is treated anonymously. The participants provided their written informed consent to participate in this study.

## Author Contributions

All authors listed have made a substantial, direct and intellectual contribution to the work, and approved it for publication.

## Conflict of Interest

The authors declare that the research was conducted in the absence of any commercial or financial relationships that could be construed as a potential conflict of interest.

## Footnotes

1 www.ledalab.de

## References

[B1] Addis M. Holbrook M. B. (2001). On the conceptual link between mass customisation and experiential consumption: an explosion of subjectivity. *J. Consum. Behav.* 1 50–66. 10.1002/cb.53

[B2] Aftanas L. I. Golocheikine S. A. (2001). Human anterior and frontal midline theta and lower alpha reflect emotionally positive state and internalized attention: high-resolution EEG investigation of meditation. *Neurosci. Lett.* 310 57–60. 10.1016/s0304-3940(01)02094-8 11524157

[B3] Ait Hammou K. Galib M. H. Melloul J. (2013). The contributions of neuromarketing in marketing research. *J. Manag. Res.* 5:20 10.5296/jmr.v5i4.4023

[B4] Alba J. W. Williams E. F. (2013). Pleasure principles: a review of research on hedonic consumption. *J. Consum. Psychol.* 23 2–18. 10.1016/j.jcps.2012.07.003

[B5] Allen C. Machleit K. Kleine S. Notani A. (2005). A place for emotion in attitude models. *J. Bus. Res.* 58 494–499. 10.1016/s0148-2963(03)00139-5

[B6] Amatulli C. De Angelis M. Donato C. (2020). An investigation on the effectiveness of hedonic versus utilitarian message appeals in luxury product communication. *Psychol. Mark.* 37 523–534. 10.1002/mar.21320

[B7] Baghi I. Antonetti P. (2017). High-fit charitable initiatives increase hedonic consumption through guilt reduction. *Eur. J. Mark.* 51 2030–2053. 10.1108/ejm-12-2016-0723

[B8] Bagozzi R. P. Gopinath M. Nyer P. U. (1999). The role of emotions in marketing. *J. Acad. Mark. Sci.* 27 184–206. 10.1177/0092070399272005

[B9] Baker J. Levy M. Grewal D. (1992). An experimental approach to making retail store environmental decisions. *J. Retail.* 68 445–460.

[B10] Barbieri R. Bianchi A. M. Triedman J. K. Mainardi L. T. Cerutti S. Saul J. P. (1997). Model dependency of multivariate autoregressive spectral analysis. *IEEE Eng. Med. Biol. Mag.* 16 74–85. 10.1109/51.620498 9313084

[B11] Barbieri R. Triedman J. Saul J. Barbieri R. Triedman J. K. Saul J. P. (2002). Heart rate control and mechanical cardiopulmonary coupling to assess central volume: a systems analysis. *Am. J. Physiol. Regul. Integr. Comp. Physiol.* 283 R1210–R1220. 12376415 10.1152/ajpregu.00127.2002

[B12] Batra R. Ahtola O. T. (1991). Measuring the hedonic and utilitarian sources of consumer attitudes. *Mark. Lett.* 2 159–170. 10.1007/bf00436035

[B13] Batra R. Stephens D. (1994). Attitudinal effects of ad-evoked moods and emotions: the moderating role of motivation. *Psychol. Mark.* 11 199–215. 10.1002/mar.4220110302

[B14] Benedek M. Kaernbach C. (2010a). A continuous measure of phasic electrodermal activity. *J. Neurosci. Methods* 190 80–91. 10.1016/j.jneumeth.2010.04.028 20451556 PMC2892750

[B15] Benedek M. Kaernbach C. (2010b). Decomposition of skin conductance data by means of nonnegative deconvolution. *Psychophysiology* 47 647–658. 20230512 10.1111/j.1469-8986.2009.00972.x PMC2904901

[B16] Berridge K. Winkielman P. (2003). What is an unconscious emotion?(The case for unconscious” liking”). *Cogn. Emot.* 17 181–211. 10.1080/02699930302289 29715719

[B17] Bettiga D. Lamberti L. (2017). Exploring the adoption process of personal technologies: a cognitive-affective approach. *J. High Technol. Manag. Res.* 28 179–187. 10.1016/j.hitech.2017.10.002

[B18] Bettiga D. Lamberti L. (2018). Exploring the role of anticipated emotions in product adoption and usage. *J. Consum. Mark.* 35 300–316. 10.1108/jcm-06-2016-1860

[B19] Bettiga D. Lamberti L. Noci G. (2017a). Do mind and body agree? Unconscious versus conscious arousal in product attitude formation. *J. Bus. Res.* 75 108–117. 10.1016/j.jbusres.2017.02.008

[B20] Bettiga D. Tacchino G. Lamberti L. Bianchi A. M. Noci G. (2017b). “Assessing consumer emotions toward new products: application of physiological and self-reported methods,” in *Proceedings of the Innovation and Product Development Management Conference (IPDMC)*, Leicester.

[B21] Bianchi A. M. Bontempi B. Cerutti S. Gianoglio P. Comi G. Natali Sora M. G. (1990). Spectral analysis of heart rate variability signal and respiration in diabetic subjects. *Med. Biol. Eng. Comp.* 28 205–211. 10.1007/bf02442668 2377001

[B22] Boucsein W. (2012). *Electrodermal Activity*, 2nd Edn Berlin: Springer Science & Business Media.

[B23] Bradley M. Lang P. (1994). Measuring emotion: the self-assessment manikin and the semantic differential. *J. Behav. Ther. Exp. Psychiatry* 25 49–59. 10.1016/0005-7916(94)90063-9 7962581

[B24] Brás S. Ferreira J. H. Soares S. C. Pinho A. J. (2018). Biometric and emotion identification: an ECG compression based method. *Front. Psychol.* 9:467 10.3389/fpsyg.2018.00467 PMC5893853 29670564

[B25] Chamberlain L. Broderick A. J. A. (2007). The application of physiological observation methods to emotion research. *Qual. Mark. Res.* 10 199–216. 10.1108/13522750710740853

[B26] Chaudhuri A. (2002). A study of emotion and reason in products and services. *J. Consum. Behav.* 1 267–279. 10.1002/cb.72

[B27] Chaudhuri A. Aboulnasr K. Ligas M. (2010). Emotional responses on initial exposure to a hedonic or utilitarian description of a radical innovation. *J. Mark. Theory Pract.* 18 339–359. 10.2753/mtp1069-6679180403

[B28] Daugherty T. Li H. Biocca F. (2008). Consumer learning and the effects of virtual experience relative to indirect and direct product experience. *Psychol. Mark.* 25 568–586. 10.1002/mar.20225

[B29] Davidson R. J. (2004). What does the prefrontal cortex “do” in affect: perspectives on frontal EEG asymmetry research. *Biol. Psychol.* 67 219–234. 10.1016/j.biopsycho.2004.03.008 15130532

[B30] Dawson M. (2011). The skin conductance response, anticipation, and decision-making. *J. Neurosci. Psychol. Econ.* 4 111–116. 10.1037/a0022619

[B31] Dawson M. Schell A. Filion D. (2007). “The electrodermal system,” in *Handbook of Psychophysiology*, eds Cacioppo J. T. Tassinary L. G. Berntson G. G. (Cambridge: Cambridge Univeristy Press), 200–223.

[B32] de Witt Huberts J. Evers C. de Ridder D. (2014). Thinking before sinning: reasoning processes in hedonic consumption. *Front. Psychol.* 5:1268 10.3389/fpsyg.2014.01268 PMC4219383 25408680

[B33] Drolet A. Williams P. Lau-Gesk L. (2007). Age-related differences in responses to affective vs. rational ads for hedonic vs. utilitarian products. *Mark. Lett.* 18 211–221. 10.1007/s11002-007-9016-z

[B34] Figner B. Murphy R. (2011). “Using skin conductance in judgment and decision making research,” in *A Handbook of Process Tracing Methods for Decision Research*, eds Schulte-Mecklenbeck M. Kühberger A. Ranyard R. (Hove: Psychology Press), 163–184.

[B35] Fiore A. M. Jin H.-J. Kim J. (2005). For fun and profit: hedonic value from image interactivity and responses toward an online store. *Psychol. Mark.* 22 669–694. 10.1002/mar.20079

[B36] Fortunato V. C. R. Giraldi J. D. M. E. De Oliveira J. H. C. (2014). A review of studies on neuromarketing: practical results, techniques, contributions and limitations. *J. Manag. Res.* 6:201 10.5296/jmr.v6i2.5446

[B37] Groeppel-Klein A. (2005). Arousal and consumer in-store behavior. *Brain Res. Bull.* 67 428–437. 10.1016/j.brainresbull.2005.06.012 16216690

[B38] Groeppel-Klein A. Baun D. (2001). The role of customers’ arousal for retail stores-results from an experimental pilot study using electrodermal activity as indicator. *Adv. Consum. Res.* 28 412–419. 10.1108/09590559810246368

[B39] Guixeres J. Bigné E. Ausín Azofra J. M. Alcañiz Raya M. Colomer Granero A. Fuentes Hurtado F. (2017). Consumer neuroscience-based metrics predict recall, liking and viewing rates in online advertising. *Front. Psychol.* 8:1808 10.3389/fpsyg.2017.01808 PMC5671759 29163251

[B40] Hair J. Sarstedt M. Ringle C. Mena J. (2012). An assessment of the use of partial least squares structural equation modeling in marketing research. *J. Acad. Mark. Sci.* 40 413–433.

[B41] Hassenzahl M. (2018). “The thing and I: understanding the relationship between user and product,” in *Funology 2. Human–Computer Interaction Series*, eds Blythe M. Monk A. (Cham: Springer), 301–313. 10.1007/978-3-319-68213-6_19

[B42] Havlena W. Holbrook M. (1986). The varieties of consumption experience: comparing two typologies of emotion in consumer behavior. *J. Consum. Res.* 13 394–404. 10.1086/209078

[B43] Hayano J. Sakakibara Y. Yamada M. Ohte N. Fujinami T. Yokoyama K. (1990). Decreased magnitude of heart rate spectral components in coronary artery disease: its relation to angiographic severity. *Circulation* 81 1217–1224. 10.1161/01.cir.81.4.1217 2317904

[B44] Henning V. Hennig-Thurau T. Feiereisen S. (2012). Giving the expectancy-value model a heart. *Psychol. Mark.* 29 765–781. 10.1002/mar.20562

[B45] Hoch S. Ha Y. (1986). Consumer learning: advertising and the ambiguity of product experience. *J. Consum. Res.* 13 221–233. 10.1086/209062

[B46] Holbrook M. B. Hirschman E. C. (1982). The experiential aspects of consumption: consumer fantasies, feelings, and fun. *J. Consum. Res.* 9 132–140. 10.1086/208906

[B47] Huber F. Eisele A. Meyer F. (2018). The role of actual, ideal, and ought self-congruence in the consumption of hedonic versus utilitarian brands. *Psychol. Mark.* 35 47–63. 10.1002/mar.21070

[B48] Ivonin L. Chang H. M. Chen W. Rauterberg M. (2013). Unconscious emotions: quantifying and logging something we are not aware of. *Pers. Ubiquitous Comput.* 17 663–673. 10.1007/s00779-012-0514-5

[B49] Johar J. S. Sirgy M. J. (1991). Value-expressive versus utilitarian advertising appeals: when and why to use which appeal. *J. Advert.* 20 23–33. 10.1080/00913367.1991.10673345

[B50] Kempf D. (1999). Attitude formation from product trial: distinct roles of cognition and affect for hedonic and functional products. *Psychol. Mark.* 16 35–50. 10.1002/(sici)1520-6793(199901)16:1<35::aid-mar3>3.0.co;2-u

[B51] Kempf D. Smith R. (1998). Consumer processing of product trial and the influence of prior advertising: a structural modeling approach. *J. Mark. Res.* 35 325–338. 10.1177/002224379803500304

[B52] Khan U. Dhar R. Wertenbroch K. (2004). *Inside Consumption: Consumer Motives, Goals, and Desires.* Abingdon: Routledge, 144–165.

[B53] Kihlstrom J. F. (1990). “The psychological unconscious,” in *Handbook of Personality. Theory and Research*, eds John O. P. Robins R. W. Pervin L. A. (New York, NY: Guilford Press), 424–442.

[B54] Kihlstrom J. F. (1992). Dissociation and dissociations: a comment on consciousness and cognition. *Conscious. Cogn.* 1 47–53. 10.1016/1053-8100(92)90044-b

[B55] Kihlstrom J. F. J. F. Mulvaney S. Tobias B. A. Tobis I. P. Eich E. (2000). “The emotional unconscious,” in *Cognition and Emotion*, eds Kihlstrom J. F. Bower G. H. Forgas J. P. Niedenthal P. M. (Oxford: Oxford University Press), 30–86.

[B56] Kim J. Morris J. (2007). The power of affective response and cognitive structure in product-trial attitude formation. *J. Advert.* 36 95–106. 10.2753/joa0091-3367360107

[B57] Kivetz R. Simonson I. (2002). Earning the right to indulge: effort as a determinant of customer preferences toward frequency program rewards. *J. Mark. Res.* 39 155–170. 10.1509/jmkr.39.2.155.19084

[B58] Klimesch W. (1999). EEG alpha and theta oscillations reflect cognitive and memory performance: a review and analysis. *Brain Res. Rev.* 29 169–195. 10.1016/s0165-0173(98)00056-3 10209231

[B59] Lang P. J. (1968). “Fear reduction and fear behavior: problems in treating a construct,” in *Research in Psychotherapy*, ed. Shlien J. M. (Washington, DC: American Psychological Association), 90–102. 10.1037/10546-004

[B60] Lerner J. Li Y. Valdesolo P. Kassam K. (2015). Emotion and decision making. *Annu. Rev. Psychol.* 66 799–823. 25251484 10.1146/annurev-psych-010213-115043

[B61] Liao C. To P. Wong Y. Palvia P. Kakhki M. D. (2016). The impact of presentation mode and product type on online impulse buying decisions. *J. Electron. Commer. Res.* 17 153–168.

[B62] Lin H. C. Bruning P. F. Swarna H. (2018a). Using online opinion leaders to promote the hedonic and utilitarian value of products and services. *Bus. Horiz.* 61 431–442. 10.1016/j.bushor.2018.01.010

[B63] Lin M. H. J. Cross S. N. Jones W. J. Childers T. L. (2018b). Applying EEG in consumer neuroscience. *Eur. J. Mark.* 52 66–91. 10.1108/ejm-12-2016-0805

[B64] Lu J. Liu Z. Fang Z. (2016). Hedonic products for you, utilitarian products for me. *Judgm. Decis. Mak.* 11 332–341.

[B65] Mainardi L. T. Bianchi A. M. Furlan R. Piazza S. Barbieri R. di Virgilio V. (1997). Multivariate time-variant identification of cardiovascular variability signals: a beat-to-beat spectral parameter estimation in vasovagal syncope. *IEEE Trans. Biomed. Eng.* 44 978–989. 10.1109/10.634650 9311167

[B66] Malhotra N. (2005). Attitude and affect: new frontiers of research in the 21st century. *J. Bus. Res.* 58 477–482. 10.1016/s0148-2963(03)00146-2

[B67] Mehrabian A. Russell J. (1974). *An Approach to Environmental Psychology.* Cambridge, MA: The MIT Press.

[B68] Mitchell A. A. (1980). The use of an information processing approach to understand advertising effects. *Adv. Consum. Res.* 7 171–176.

[B69] Mittal B. Lee M.-S. (1989). A causal model of consumer involvement. *J. Econ. Psychol.* 10 363–389. 10.1016/0167-4870(89)90030-5

[B70] Neelamegham R. Jain D. (1999). Consumer choice process for experience goods: an econometric model and analysis. *J. Mark. Res.* 36 373–386. 10.2307/3152083

[B71] Oatley K. (1992). *Best Laid Schemes: The Psychology of the Emotions.* Cambridge: Cambridge University Press.

[B72] O’Brien H. L. O’Brien H. (2010). The influence of hedonic and utilitarian motivations on user engagement: the case of online shopping experiences. *Interact. Comput.* 22 344–352. 10.1016/j.intcom.2010.04.001

[B73] Öhman A. (1986). Face the beast and fear the face: animal and social fears as prototypes for evolutionary analyses of emotion. *Psychophysiology* 23 123–145. 10.1111/j.1469-8986.1986.tb00608.x 3704069

[B74] Pan J. Tompkins W. J. (1985). A real-time QRS detection algorithm. *IEEE Trans. Biomed. Eng.* 32 230–236. 10.1109/tbme.1985.325532 3997178

[B75] Pham M. T. (1998). Representativeness, relevance, and the use of feelings in decision making. *J. Consum. Res.* 25 144–159. 10.1086/209532

[B76] Podsakoff P. MacKenzie S. Lee J. Podsakoff N. (2003). Common method biases in behavioral research: a critical review of the literature and recommended remedies. *J. Appl. Psychol.* 88 879–903. 10.1037/0021-9010.88.5.879 14516251

[B77] Ren J. Nickerson J. V. (2019). Arousal, valence, and volume: how the influence of online review characteristics differs with respect to utilitarian and hedonic products. *Eur. J. Inform. Syst.* 28 272–290. 10.1080/0960085x.2018.1524419

[B78] Robinson M. D. Clore G. L. (2002). Belief and feeling: evidence for an accessibility model of emotional self-report. *Psychol. Bull.* 128 934–960. 10.1037/0033-2909.128.6.934 12405138

[B79] Rossiter J. R. Percy L. Donovan R. J. (1991). A better advertising planning grid. *J. Advert. Res.* 31 11–21.

[B80] Sequeira H. Hot P. Silvert L. Delplanque S. (2009). Electrical autonomic correlates of emotion. *Int. J. Psychophysiol.* 71 50–56. 10.1016/j.ijpsycho.2008.07.009 18723054

[B81] Sharma P. Chan R. Y. K. (2017). Exploring the role of attitudinal functions in counterfeit purchase behavior via an extended conceptual framework. *Psychol. Mark.* 34 294–308. 10.1002/mar.20989

[B82] Smith R. Lane R. D. (2016). Unconscious emotion: a cognitive neuroscientific perspective. *Neurosci. Biobehav. Rev.* 69 216–238. 10.1016/j.neubiorev.2016.08.013 27522011

[B83] Strahilevitz M. Myers J. (1998). Donations to charity as purchase incentives: how well they work may depend on what you are trying to sell. *J. Consum. Res.* 24 434–446. 10.1086/209519

[B84] Sung B. Wilson N. J. Yun J. H. Lee E. J. (2019). What can neuroscience offer marketing research? *Asia Pac. J. Mark. Logist.* 32 1089–1111. 10.1108/apjml-04-2019-0227

[B85] Vecchiato G. Astolfi L. De Vico Fallani F. Cincotti F. Mattia D. Salinari S. (2010). Changes in brain activity during the observation of TV commercials by using EEG, GSR and HR measurements. *Brain Topogr.* 23 165–179. 10.1007/s10548-009-0127-0 20033272

[B86] Vecchiato G. Cherubino P. Maglione A. G. Hu S. Wei D. Colosimo A. (2012). Comparison of cognitive and emotional cerebral variables in Eastern subjects watching TV advertisements: a case study. *Int. J. Bioelectromagn.* 14 127–132.

[B87] Vecchiato G. Toppi J. Astolfi L. Fallani F. F. D. V. De Vico Fallani F. Cincotti F. (2011). Spectral EEG frontal asymmetries correlate with the experienced pleasantness of TV commercial advertisements. *Med. Biol. Eng. Comput.* 49 579–583. 10.1007/s11517-011-0747-x 21327841

[B88] Venkatraman V. Dimoka A. Pavlou P. A. Vo K. Hampton W. Bollinger B. (2015). Predicting advertising success beyond traditional measures: new insights from neurophysiological methods and market response modeling. *J. Mark. Res.* 52 436–452. 10.1509/jmr.13.0593

[B89] Vila-López N. Küster-Boluda I. (2018). Commercial versus technical cues to position a new product: do hedonic and functional/healthy packages differ? *Soc. Sci. Med.* 198 85–94. 10.1016/j.socscimed.2017.12.018 29289931

[B90] Ward J. Barnes J. (2001). Control and affect: the influence of feeling in control of the retail environment on affect, involvement, attitude, and behavior. *J. Bus. Res.* 54 139–144. 10.1016/s0148-2963(99)00083-1

[B91] Webster J. Ho H. (1997). Audience engagement in multimedia presentations. *ACM SIGMIS Database* 28 63–77. 10.1145/264701.264706

[B92] Wertenbroch K. Dhar R. (2000). Consumer choice between hedonic and utilitarian goods. *J. Mark. Res.* 37 60–71. 10.1509/jmkr.37.1.60.18718

[B93] Woods W. (1960). Psychological dimensions of consumer decision. *J. Mark.* 24 15–19. 10.1177/002224296002400303

[B94] Yang Z. Wu Y. Lu C. Tu Y. (2020). Effects of paid search advertising on product sales: a Chinese semantic perspective. *J. Mark. Manag.* 1–24. 10.1080/0267257x.2020.1765001

[B95] Youn S. Sun T. Wells W. D. Zhao X. (2001). Commercial liking and memory: moderating effects of product categories. *J. Advert. Res.* 41 7–13. 10.2501/jar-41-3-7-13

[B96] Zaichkowsky J. L. (1985). Measuring the involvement construct. *J. Consum. Res.* 12 341–352. 10.1086/208520

[B97] Zaltman G. (1997). Rethinking market research: putting people back in. *J. Mark. Res.* 34 424–437. 10.2307/3151962

[B98] Zheng Y. Kivetz R. (2009). The differential promotion effectiveness on hedonic versus utilitarian products. *Adv. Consum. Res.* 36:565.

